# Supplementary figures and images for: Investigating the impact of reference assembly choice on genomic analyses in a cattle breed
Source: BMC Genomics. 2021 May 19;22:363. doi: 10.1186/s12864-021-07554-w (PMC8132449; doi:10.1186/s12864-021-07554-w)

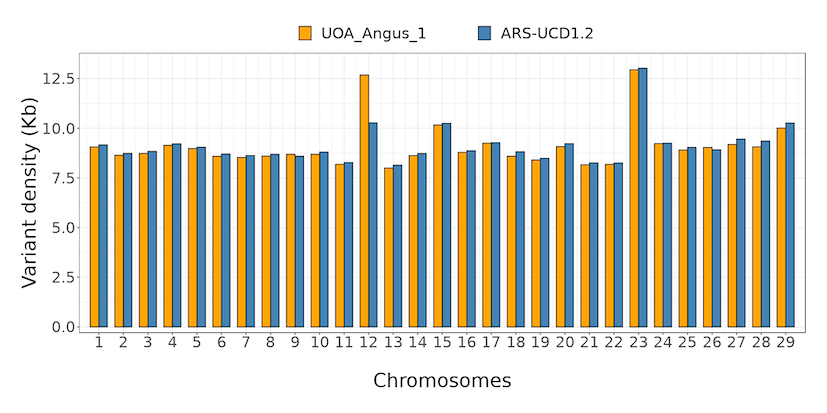

Supplement: Supplementary file 4 — Additional file 4 Figure S1: Variant density of the autosomes for both assemblies. Number of variants detected per kilo base pair (Kb) along autosomal sequences of 161 BSW samples when aligned to the ARS-UCD1.2 (blue) and UOA_Angus_1 (orange) assembly. [file 12864_2021_7554_MOESM4_ESM.png]

**A**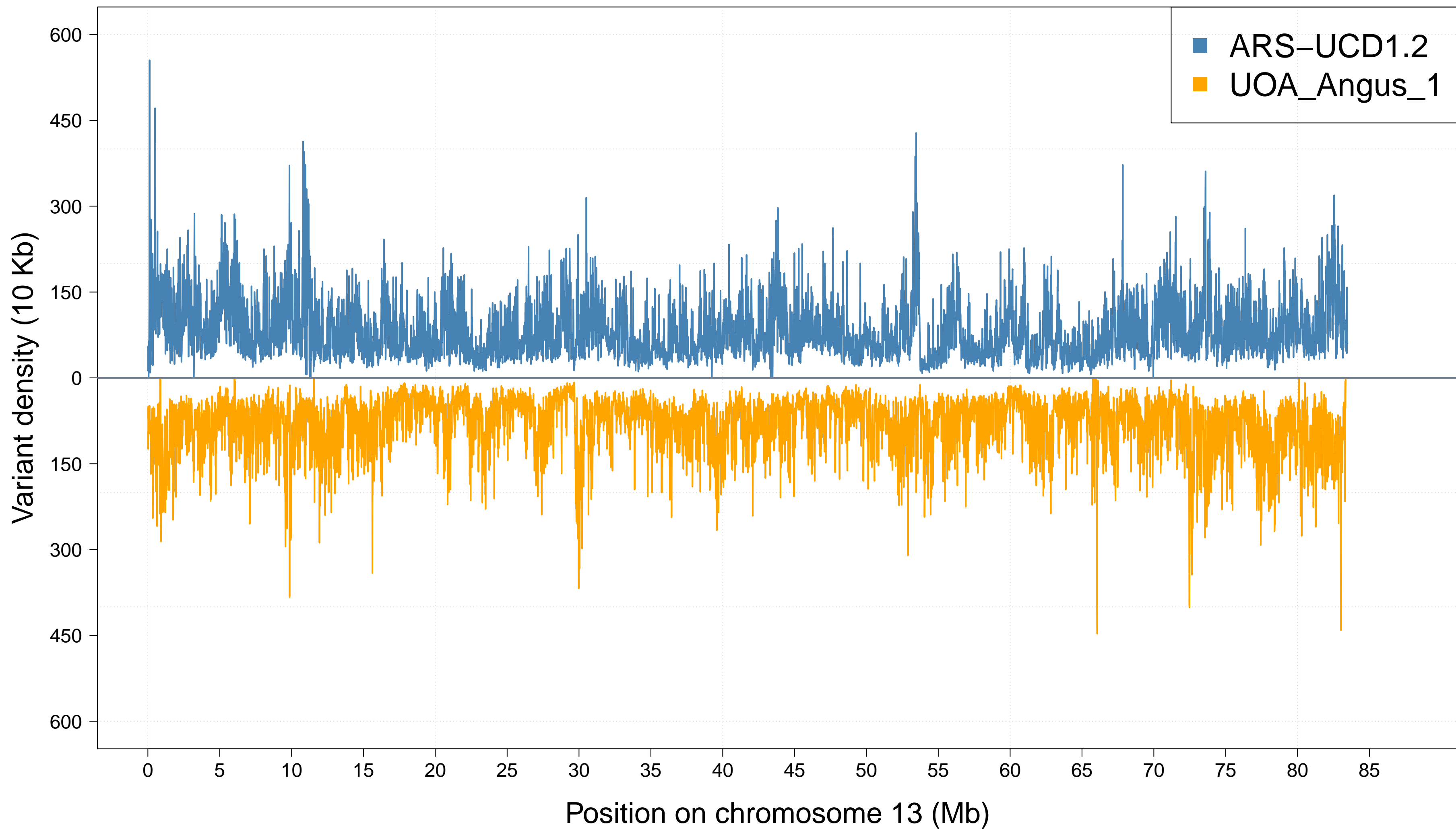

**B**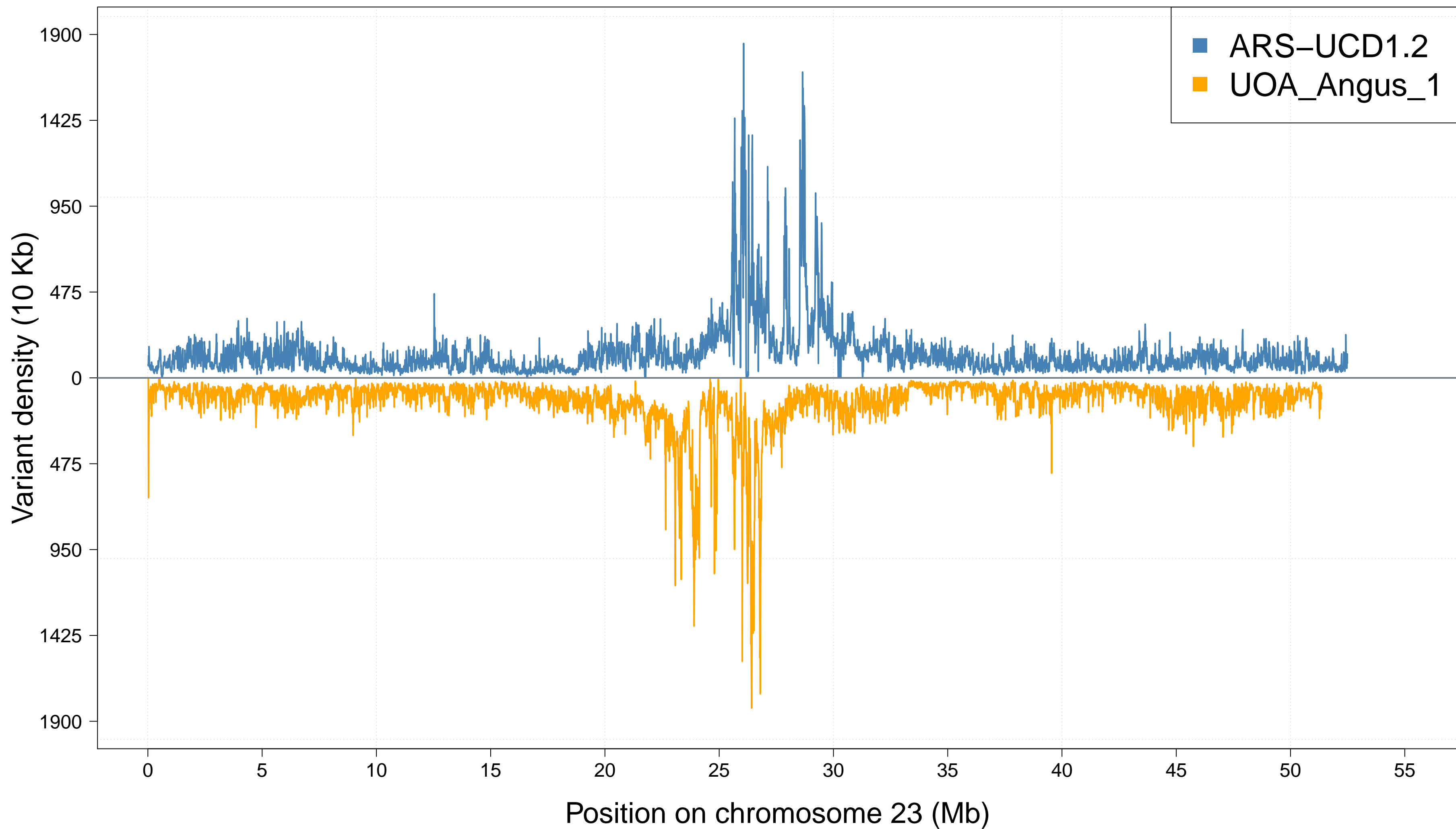

Supplement: Supplementary file 5 — Additional file 5 Figure S2: Density of variants across chromosomes 13 and 23. The number of variants is shown within non-overlapping windows of 10 Kb for chromosome 13 (A) and 23 (B). The x-axis indicates the length of the chromosome (in Mb). The number of variants within each 10 Kb window is shown on the y-axis. Assembly ARS-UCD1.2 is displayed in the top panel (blue) and assembly UOA_Angus_1 is displayed as a mirror image in the bottom panel (orange). [file 12864_2021_7554_MOESM5_ESM.pdf]

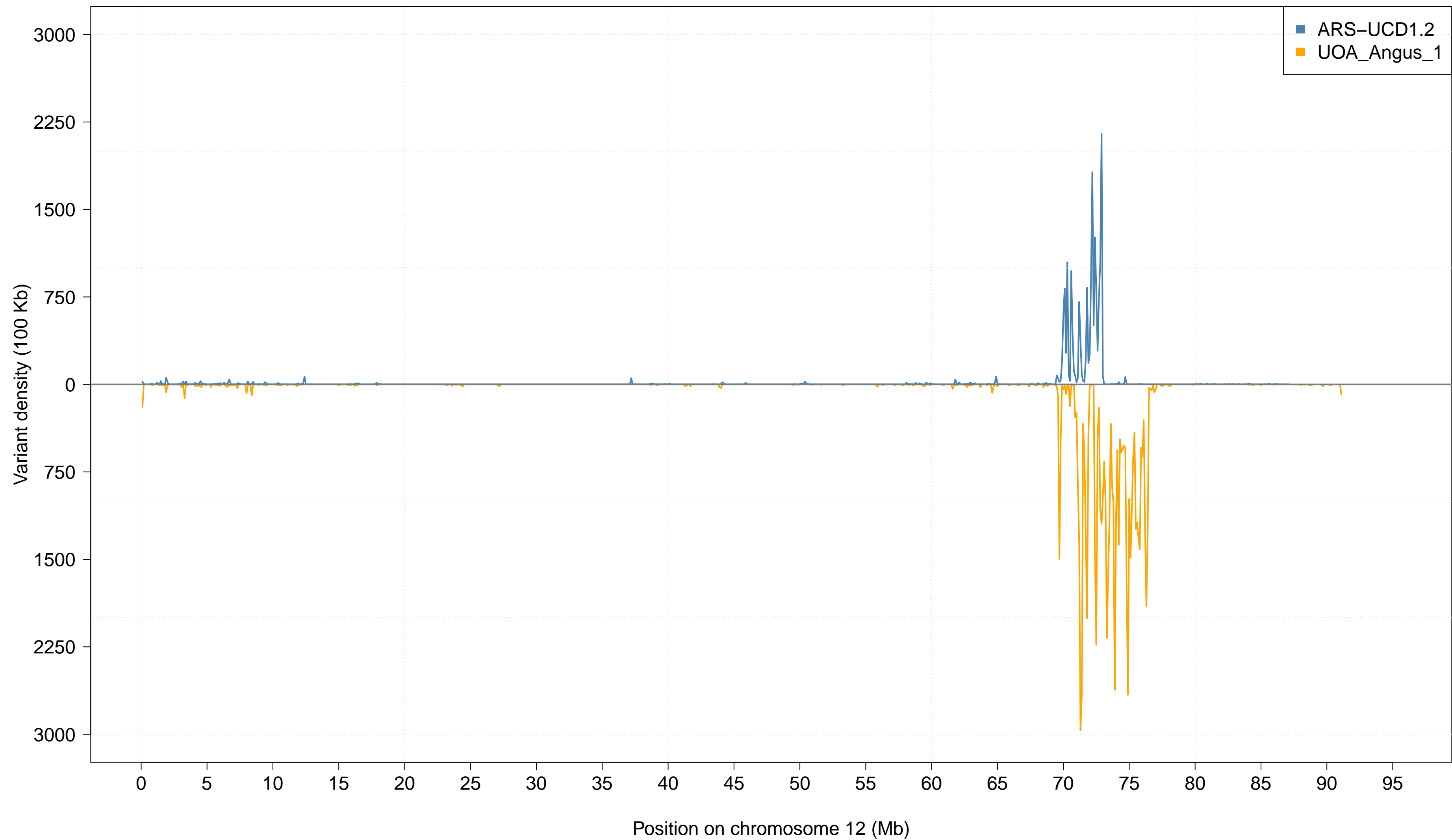

Supplement: Supplementary file 8 — Additional file 8 Figure S3: Density of variants deviating from Hardy-Weinberg proportion for chromosome 12. The number of variants differing from Hardy-Weinberg proportion are plotted as non-overlapping windows of 10 Kb along the autosomal sequence. The y-axis relates the variant density, number of variants per 100 Kb, for each 10-Kb-windows. [file 12864_2021_7554_MOESM8_ESM.pdf]

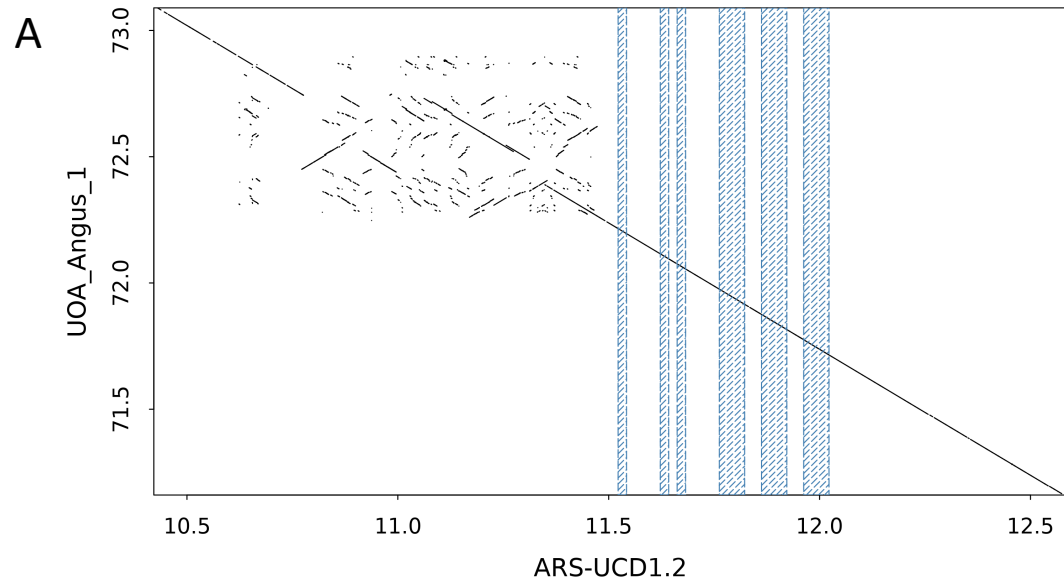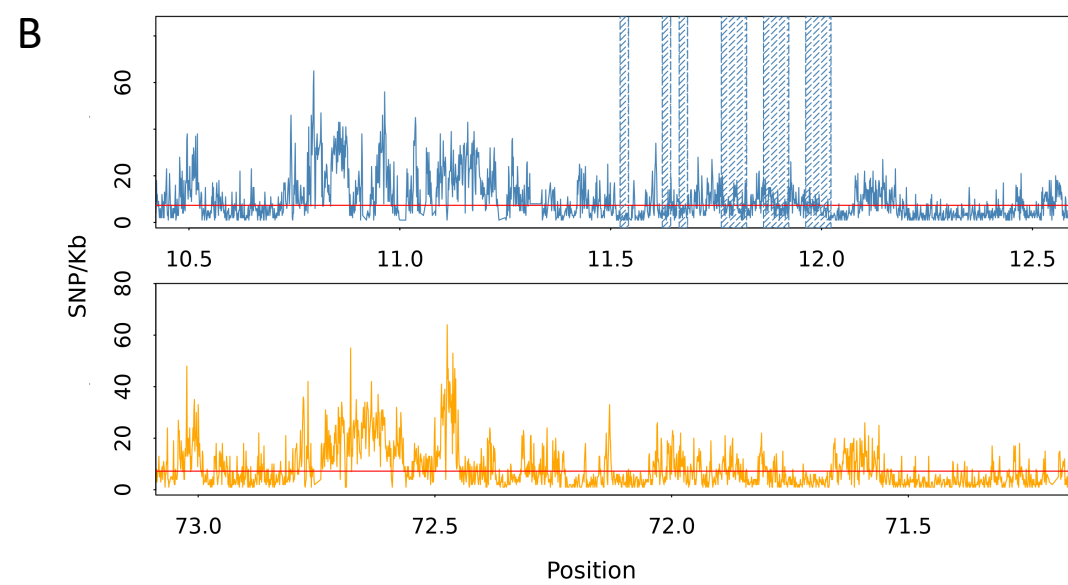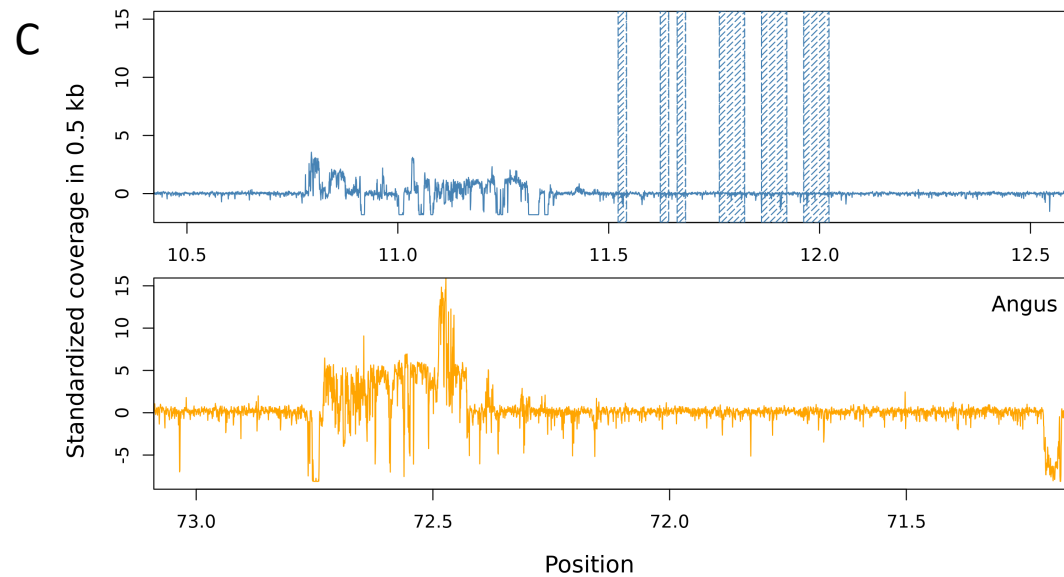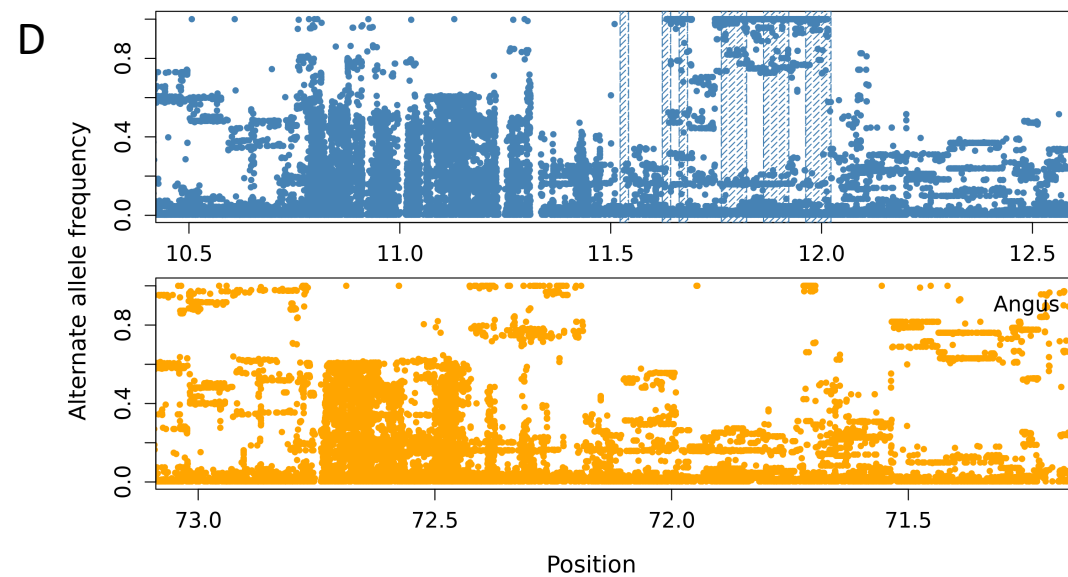

Supplement: Supplementary file 12 — Additional file 12 Figure S4: Selective sweeps on chromosome 13. Chromosome 13 region in ARS-UCD1.2 from 10,501,688 - 12,506,844 Mb and corresponding region on UOA_Angus_1 between 71,231,671 - 73,018,009 Mb with highlighted six selective sweep region from 11.5 Mb to 12 Mb. (A) Dot plot between the two assemblies, (B) SNP density per Kb (red line represents the average SNP density/chromosome), (C) Standardized coverage per 0.5 Kb, (D) Alternate allele frequency of each SNP (each dot is per SNP). [file 12864_2021_7554_MOESM12_ESM.pdf]

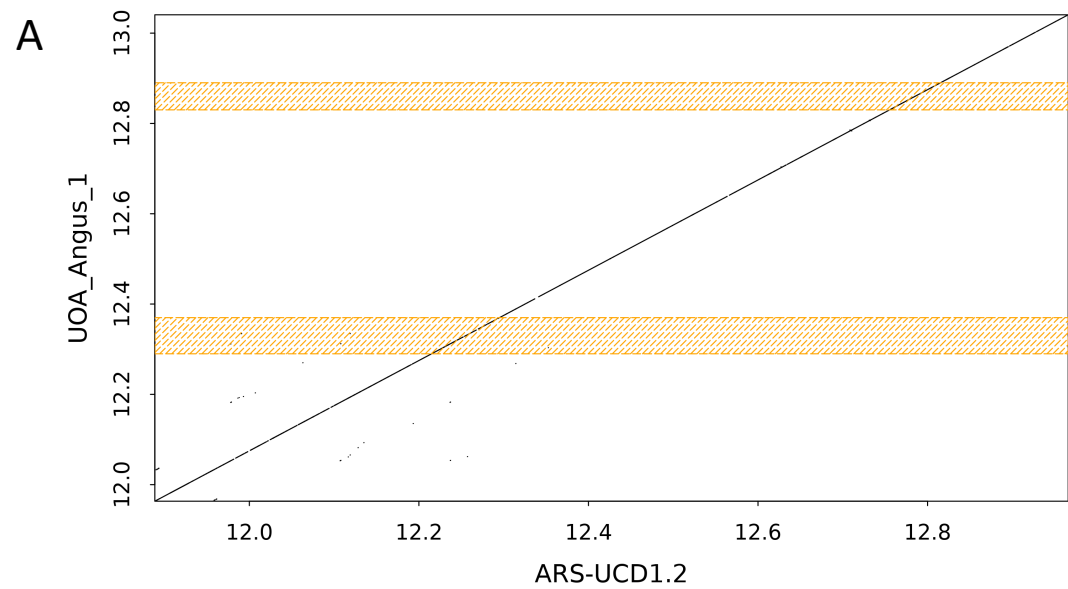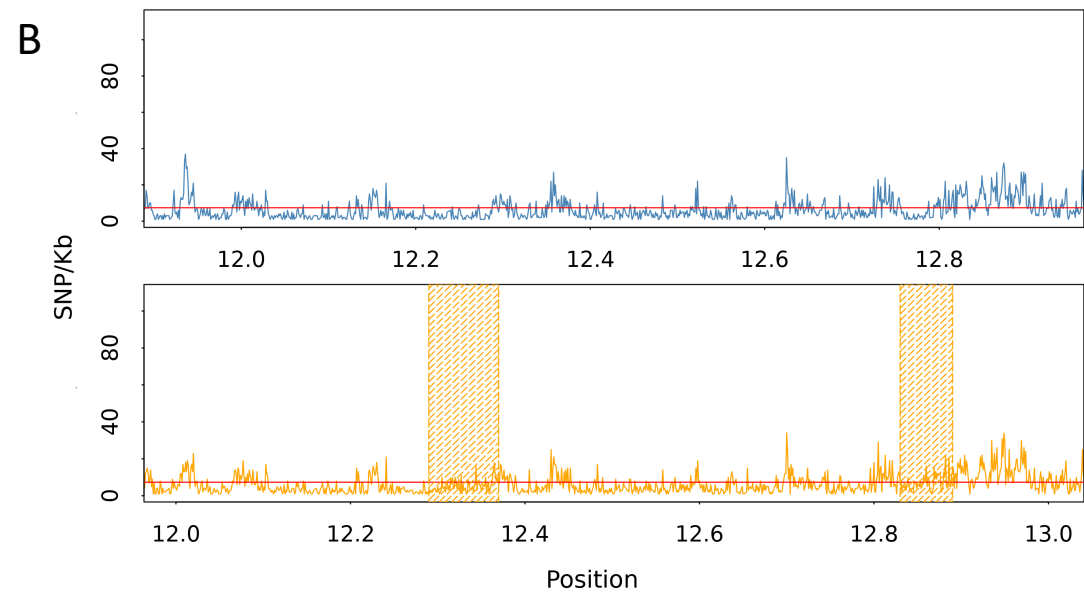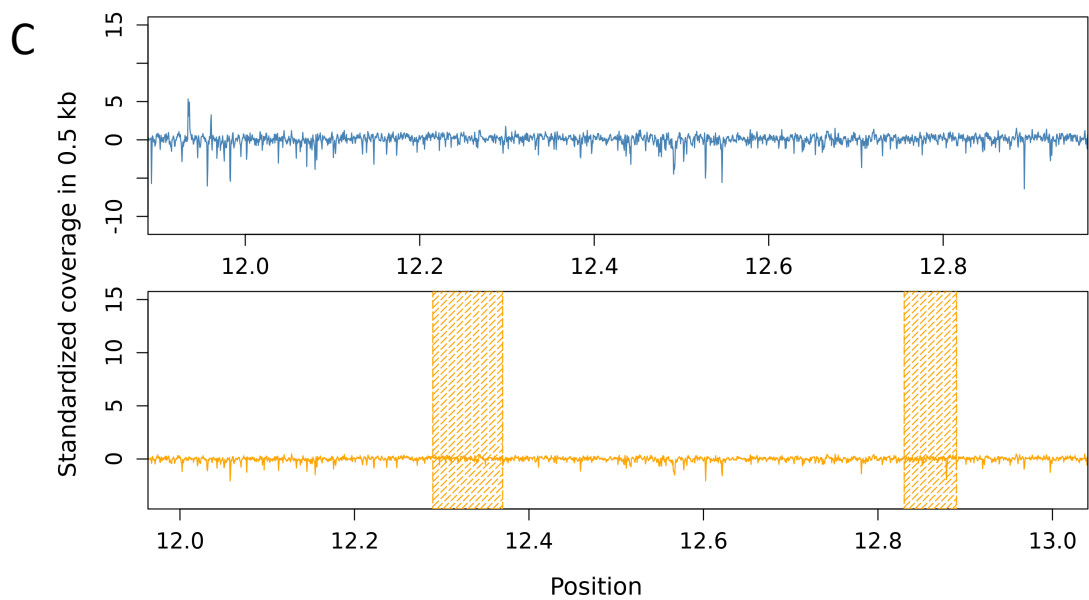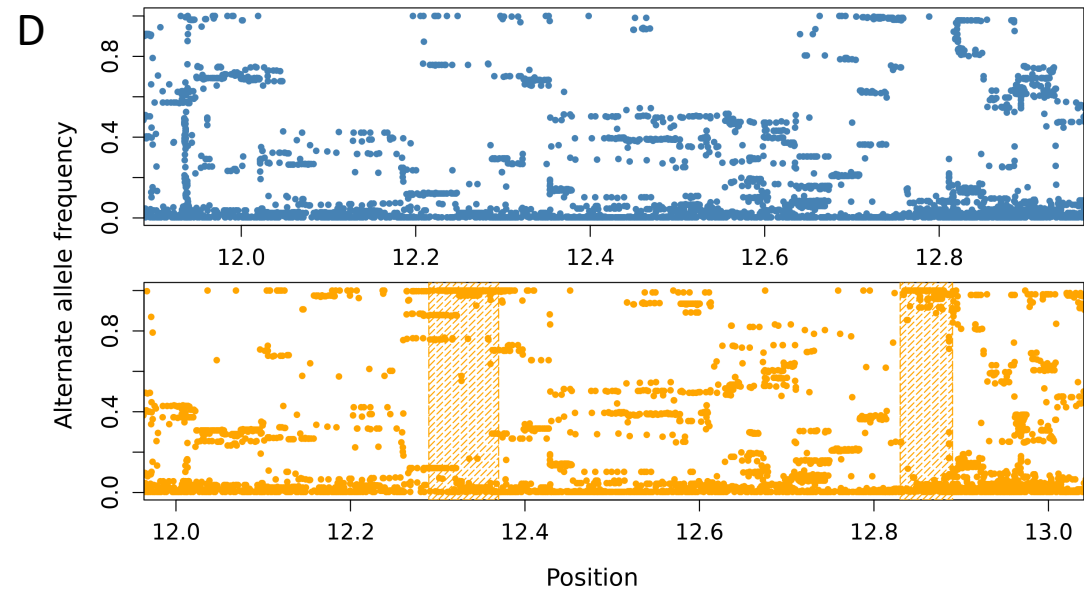

Supplement: Supplementary file 13 — Additional file 13 Figure S5: Selective sweeps on chromosome 22. Chromosome 22 region in ARS-UCD1.2 from 11,928,425 - 12,925,926 Mb and corresponding region on UOA_Angus_1 between 12,003,259 - 13,000,720 Mb with highlighted two selective sweep region. (A) Dot plot between the two assemblies, (B) SNP density per Kb (red line represents the average SNP density/chromosome), (C) Standardized coverage per 0.5 Kb, (D) Alternate allele frequency of each SNP (where each dot is per SNP). [file 12864_2021_7554_MOESM13_ESM.pdf]

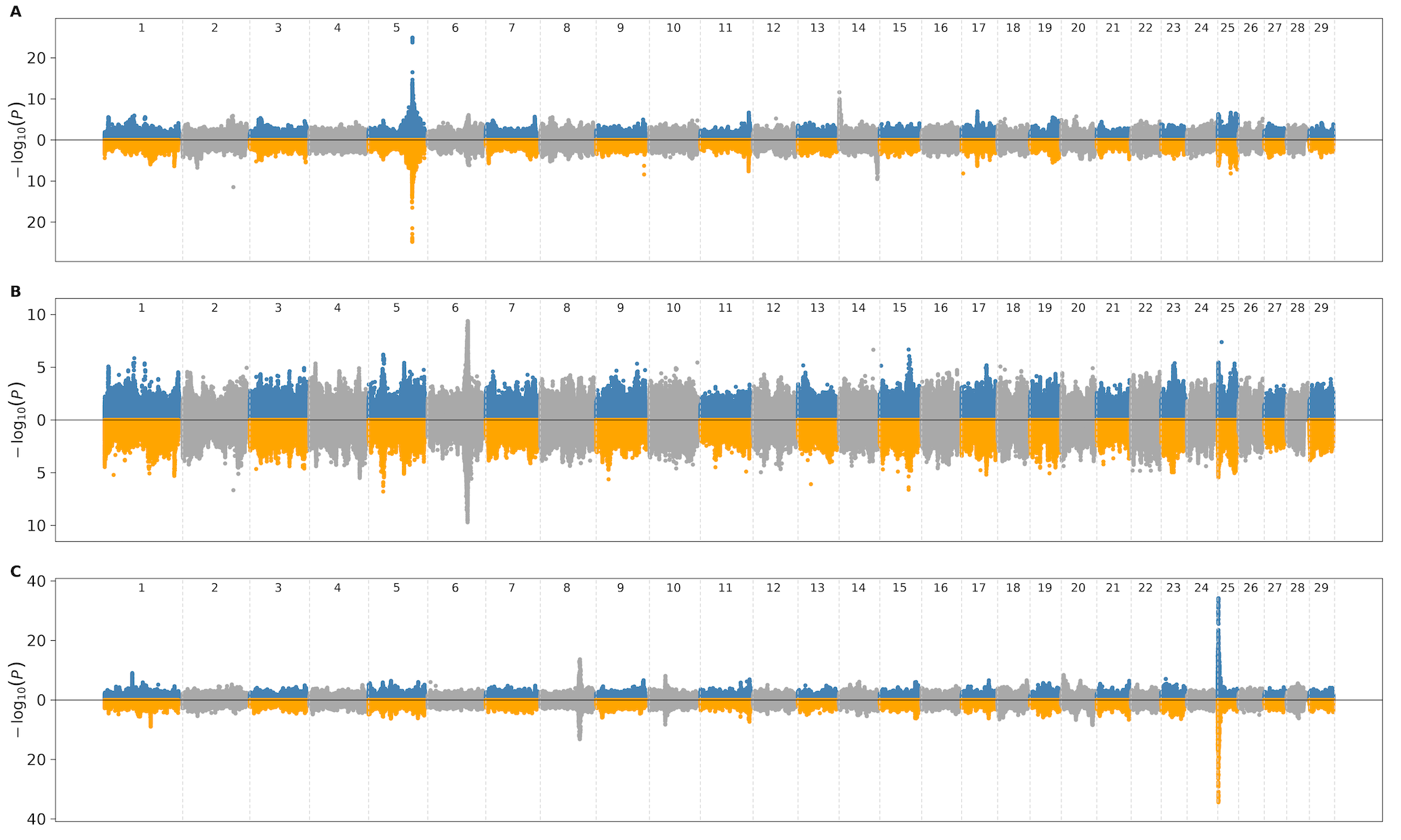

Supplement: Supplementary file 14 — Additional file 14 Figure S6: Genome Wide Association Study (GWAS). Manhattan plots showing association of sequence variants - imputed using ARS-UCD1.2 (blue and grey) and UOA_Angus_1 (orange and grey) - with fat yield (A), protein yield (B) and stature (C). [file 12864_2021_7554_MOESM14_ESM.tiff]
